# Comparison and evaluation of two different methods to establish the cigarette smoke exposure mouse model of COPD

**DOI:** 10.1038/s41598-017-15685-y

**Published:** 2017-11-13

**Authors:** Jiaze Shu, Defu Li, Haiping Ouyang, Junyi Huang, Zhen Long, Zhihao Liang, Yuqin Chen, Yiguan Chen, Qiuyu Zheng, Meidan Kuang, Haiyang Tang, Jian Wang, Wenju Lu

**Affiliations:** 1 State Key Laboratory of Respiratory Diseases, Guangzhou Institute of Respiratory Disease, The First Affiliated Hospital of Guangzhou Medical University, 151 Yanjiang Road, Guangzhou, Guangdong, 510120 P.R. China; 2 Division of Translational and Regenerative Medicine, Department of Medicine and Department of Physiology, The University of Arizona College of Medicine, Tucson, Arizona United States

## Abstract

Animal model of cigarette smoke (CS) –induced chronic obstructive pulmonary disease (COPD) is the primary testing methodology for drug therapies and studies on pathogenic mechanisms of disease. However, researchers have rarely run simultaneous or side-by-side tests of whole-body and nose-only CS exposure in building their mouse models of COPD. We compared and evaluated these two different methods of CS exposure, plus airway Lipopolysaccharides (LPS) inhalation, in building our COPD mouse model. Compared with the control group, CS exposed mice showed significant increased inspiratory resistance, functional residual capacity, right ventricular hypertrophy index, and total cell count in BALF. Moreover, histological staining exhibited goblet cell hyperplasia, lung inflammation, thickening of smooth muscle layer on bronchia, and lung angiogenesis in both methods of CS exposure. Our data indicated that a viable mouse model of COPD can be established by combining the results from whole-body CS exposure, nose-only CS exposure, and airway LPS inhalation testing. However, in our study, we also found that, given the same amount of particulate intake, changes in right ventricular pressure and intimal thickening of pulmonary small artery are a little more serious in nose-only CS exposure method than changes in the whole-body CS exposure method.

## Introduction

Chronic Obstructive Pulmonary Disease (COPD), a common preventable and treatable disease, is characterized by persistent airflow limitation, is usually progressive, and is associated with an enhanced chronic inflammatory response to noxious particles or gases in the airways and the lung. Exacerbations and comorbidities contribute to the overall severity in individual patients^1^. COPD, which is the fourth leading cause of death in the world, now becomes an increasingly important public health challenge. COPD causes fateful chronic morbidity and mortality throughout the world, with many suffering for several decades, and dying prematurely from it, or its complications. Throughout the world, cigarette smoking is the most common risk factor for COPD^1^. Cigarette smokers are far more susceptible to the respiratory symptoms and lung function abnormalities than non-smokers^2^.

Pulmonary hypertension (PH) may develop late in the progression of COPD, and its severity is an important factor impacting on the prognosis of patients^3^. As one of the common complications of COPD, PH is mainly due to hypoxic vasoconstriction of small pulmonary arteries, eventually resulting in pathologic changes in structure, including intimal hyperplasia, and later, smooth muscle hypertrophy/hyperplasia^4^. COPD morbidity and mortality are significantly increased by the common occurrence of pulmonary hypertension associated with cigarette smoke^5^. The loss of the pulmonary capillary bed in emphysema may also contribute to the increased pressure in pulmonary circulation^6^. Progressive pulmonary hypertension may lead to right ventricular hypertrophy, and ultimately to right ventricular failure and even death^7^.

Cigarette smoke (CS) is the main preventable cause of COPD, resulting in progressive proteolytic inflammatory and pulmonary hypertension. Currently, animal CS exposure models are the most appropriate method for studying COPD. There are several exposure systems being used to investigate the effects of CS exposure in such models^8–10^. The smoking machines used in animal models feature systems for both nose-only and whole-body exposures. However, compared with whole-body CS exposure models, the nose-only CS exposure models are more controllable and reproducible, because they avoid the problem, in whole-body exposure studies, of potential increased uptake of nicotine, tar and/or other cigarette substances, as the animals clean their fur coats^8,9^.

LPS is a major proinflammatory glycolipid component of the gram-negative bacteria cell wall. Establishing a standardized COPD animal model consistent with the clinical situation is essential for carrying out this research. Sun^11^ established a COPD rat model which well recapitulates the pathophysiology and development of human COPD, by implementing a method which combines cigarette smoke exposure and trachea injection of LPS, to simulate smoking and infection, two most important incentives to developing human COPD. This combination of LPS and CS aimed to simulate the acute exacerbation of COPD symptoms seen in patients with infection, who then suffer a sharp decline in pulmonary function.

Until now, only a few studies have used a nose-only exposure system to establish COPD models, and most of them focused mainly on the effects of CS exposure on lung function and airway inflammation^12,13^. However, to our knowledge, most researchers rarely combine whole-body and nose-only testing to build their mouse models. Exactly how the pulmonary vascular changes develop over time, and whether these changes are related to lung damage and airway inflammation in the nose-only CS exposure model, is still unknown. Therefore, the aim of our study was to evaluate the combination of these two different CS exposure models, plus airway Lipopolysaccharides (LPS) inhalation, to establish an improved COPD mouse model.

## Results

### Lung function measurement

**Figure 1:**
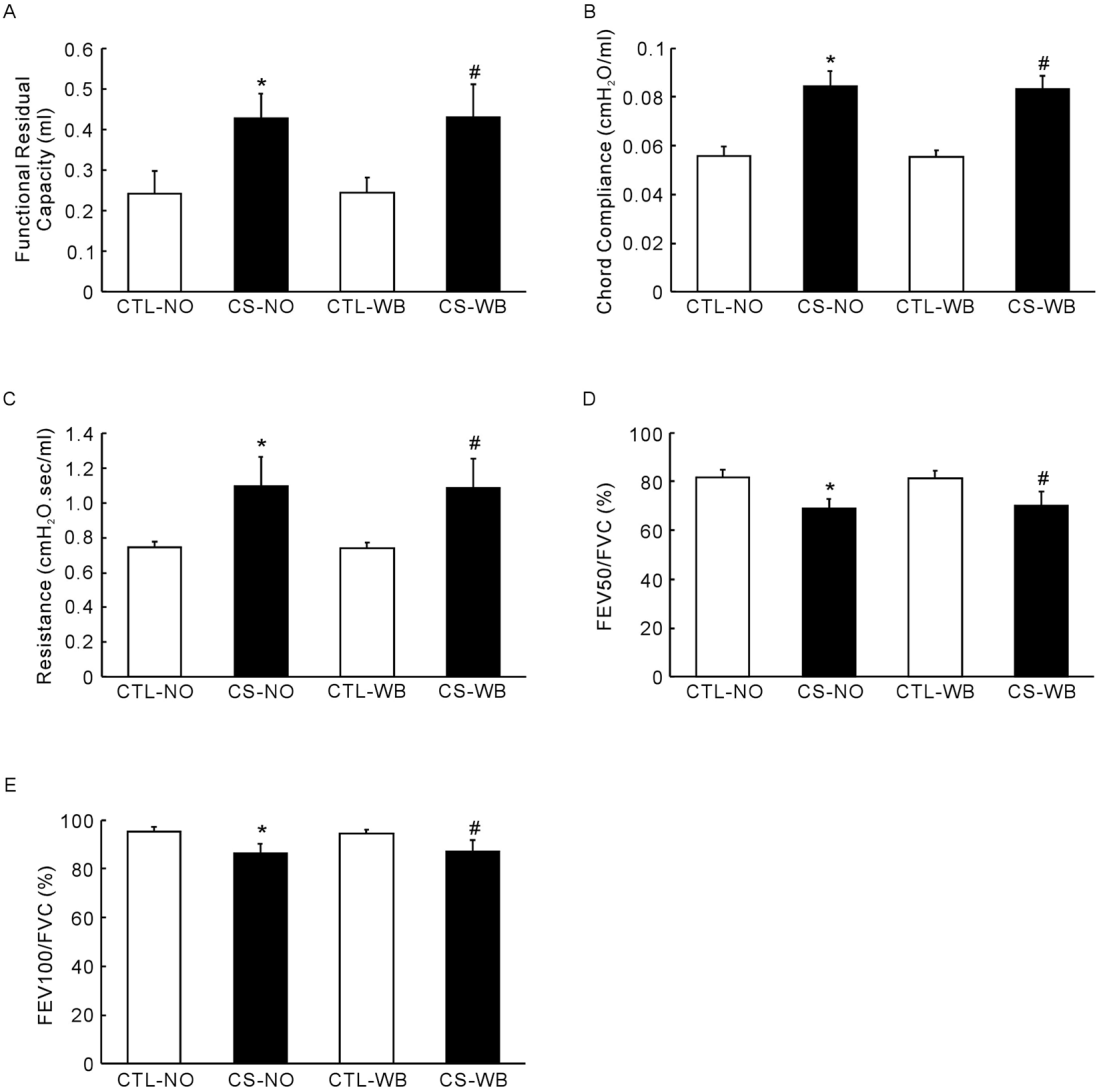
Lung function measurement, including FRC (**A**), Chord compliance (**B**), inspiratory resistance (**C**), FEV_50_/FVC (**D**) and FEV_100_/FVC (**E**), following the group of nose-only CS exposure (CS-NO) or air control (CTL-NO) and the group of whole-body CS exposure (CS-WB) or air control (CTL-WB). Data are presented as mean ± SD (n = 6–9), *P < 0.01 for CS-NO group vs. CTL-NO group, ^#^ *P* < 0.01 for CS-WB group vs. CTL-WB group.

As is shown in the Fig. 1, compared with the control group, nose-only CS exposure and whole-body CS exposure caused a significant increase in FRC (*P* < 0.01), C-chord (*P* < 0.01), inspiratory resistance (*P* < 0.01), and caused a decrease in FEV_50_/FVC (*P* < 0.01) and FEV_100_/FVC (*P* < 0.01). However, there is no significant difference between the nose-only CS exposure group and the whole-body CS exposure group in the lung function measurements above.

### Cell composition and number in BALF

**Figure 2:**
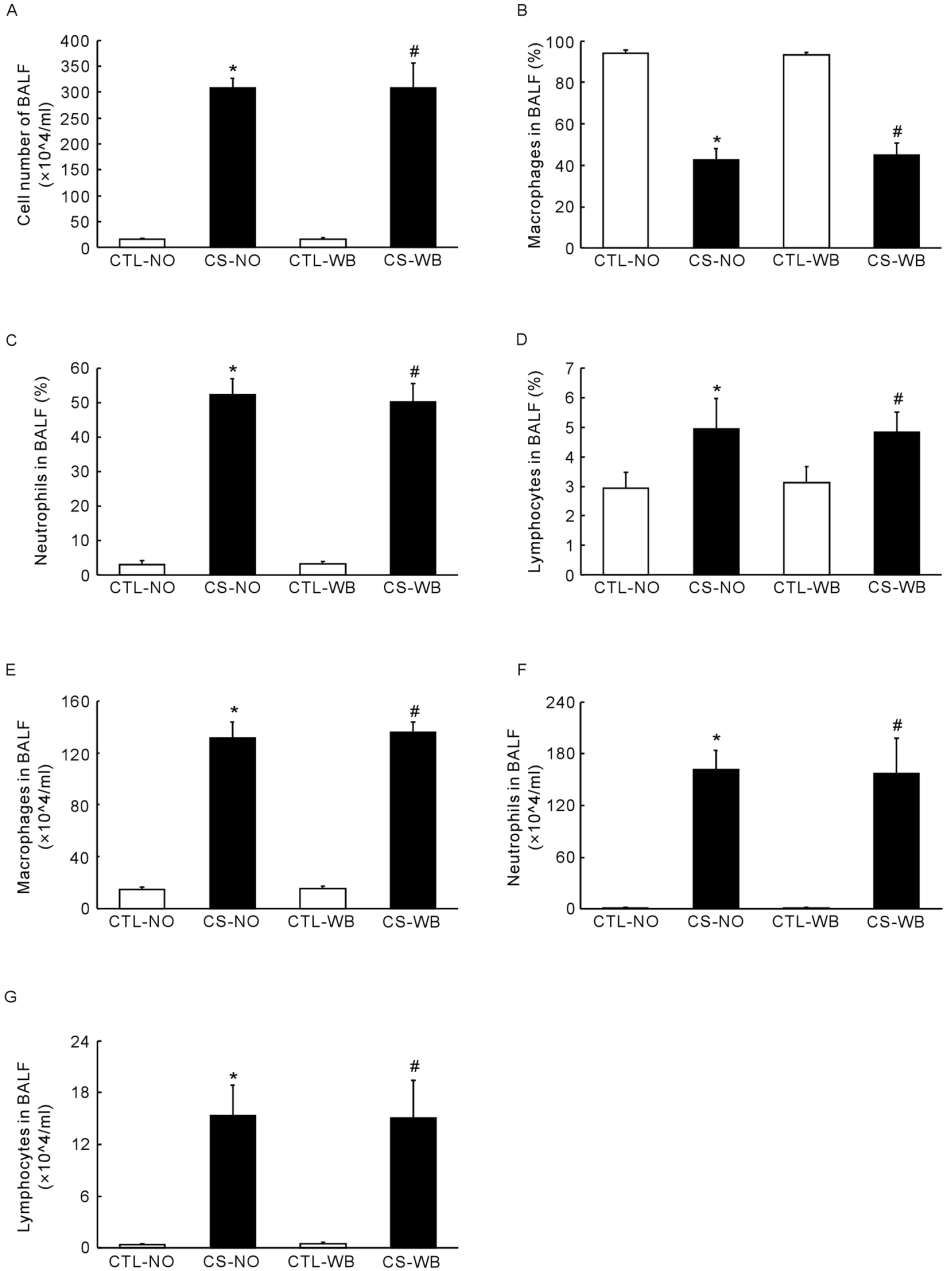
Number of total cells in BALF (**A**), the proportion of macrophages (**B**), neutrophils (**C**) and lymphocytes (**D**), the number of macrophages (**E**), neutrophils (**F**) and lymphocytes (**G**) in the total BALF cells, following the group of nose-only CS exposure (CS-NO) or air control (CTL-NO) and the group of whole-body CS exposure (CS-WB) or air control (CTL-WB). Data are presented as mean ± SD (n = 6–9), *P < 0.01 for CS-NO group vs. CTL-NO group, ^#^ *P* < 0.01 for CS-WB group vs. CTL-WB group.

The number of total cells in BALF was significantly increased (*P* < 0.01) in the nose-only CS exposure group and the whole-body CS exposure group, compared with their respective air-exposure control groups (Fig. 2). The proportion of neutrophils (*P* < 0.01) and lymphocytes (*P* < 0.01) in BALF increased, while the proportion of macrophages decreased (*P* < 0.01) in the two CS exposure groups, compared with the control groups. The cell number of neutrophils (*P* < 0.01), lymphocytes (*P* < 0.01) and macrophages (*P* < 0.01) increased in BALF in both the nose-only CS exposure group and the whole-body CS exposure group, compared with their respective air-exposure groups. But similarly, no significant difference was found between two the CS exposure groups in cell composition and number in BALF.

### Protein levels of IL6 and KC in BALF

**Figure 3:**
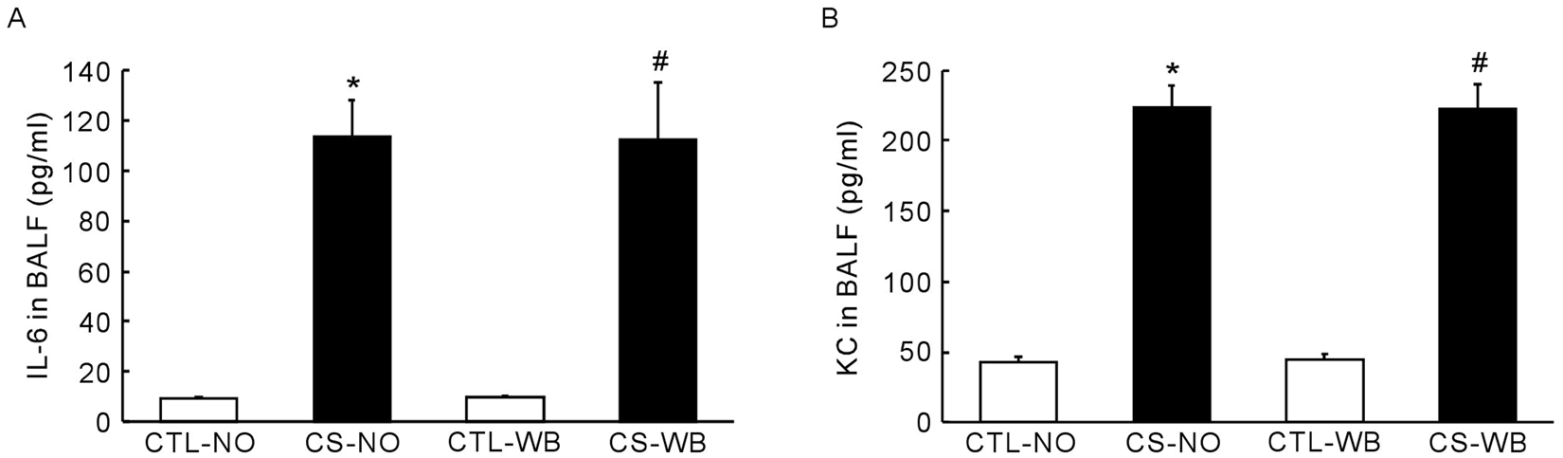
The protein levels of IL6 (**A**) and KC (**B**) in BALF, following the group of nose-only CS exposure (CS-NO) or air control (CTL-NO) and the group of whole-body CS exposure (CS-WB) or air control (CTL-WB). Data are presented as mean ± SD (n = 6–9), *P < 0.01 for CS-NO group vs. CTL-NO group, ^#^ *P* < 0.01 for CS-WB group vs. CTL-WB group.

Nose-only CS exposure and whole-body CS exposure both induced a significant increase in the level of IL6 (*P* < 0.01) and KC (*P* < 0.01) in BALF, compared with the control group mice (Fig. 3). However, there is no significant difference in the level of IL6 and KC between the two CS exposure groups.

### Right ventricular pressure and hypertrophy index

**Figure 4:**
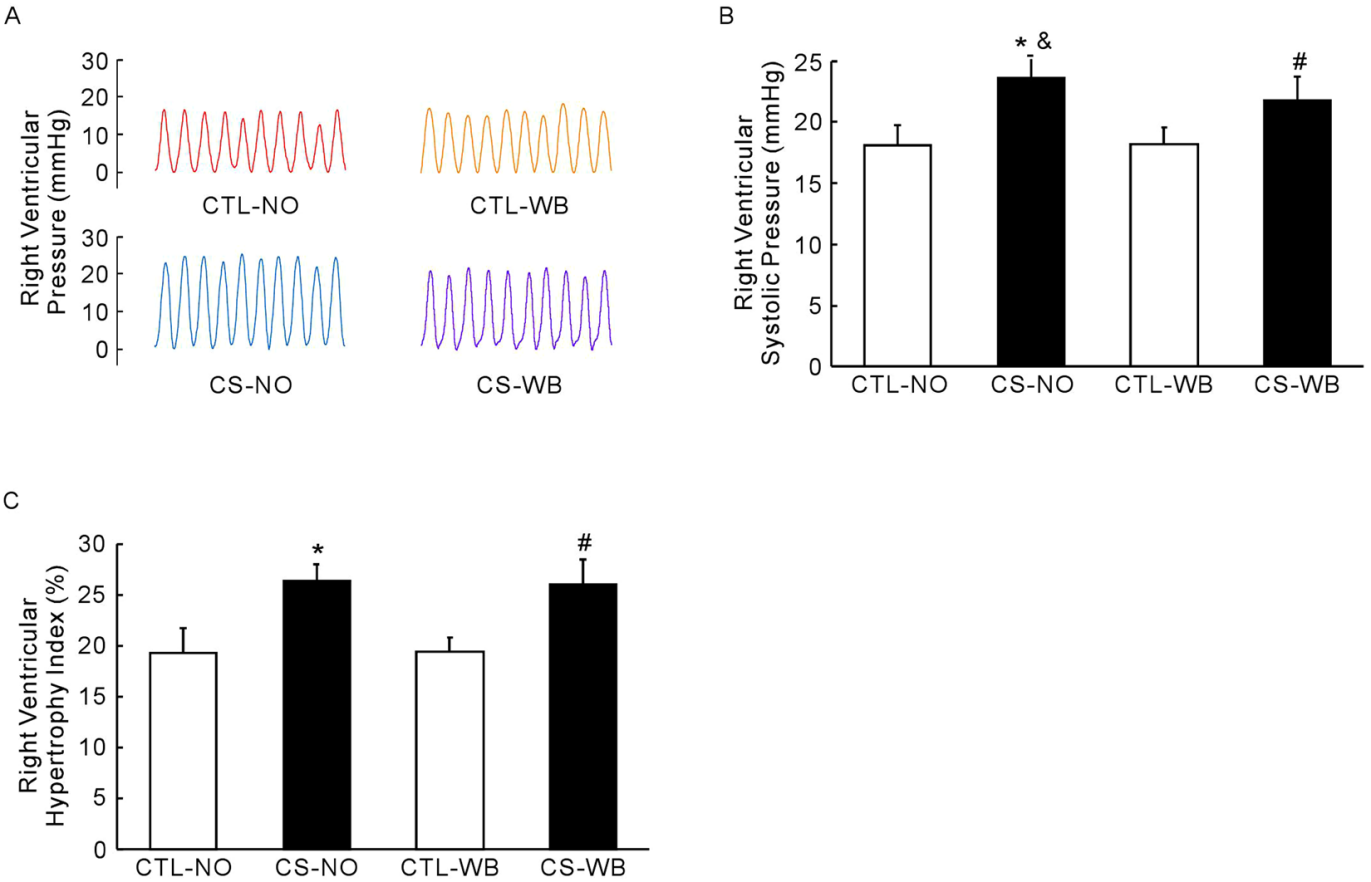
Right ventricular pressure and hypertrophy index, including representative traces of right ventricular pressure of each group of animals (**A**), right ventricular systolic pressure (**B**) and right ventricular hypertrophy index (**C**), following the group of nose-only CS exposure (CS-NO) or air control (CTL-NO) and the group of whole-body CS exposure (CS-WB) or air control (CTL-WB). Data are presented as mean ± SD (n = 6–9), *P < 0.01 for CS-NO group vs. CTL-NO group, ^#^ *P* < 0.01 for CS-WB group vs. CTL-WB group, ^&^ *P* < 0.05 for CS-NO group vs. CS-WB group.

Compared with the control groups, nose-only CS exposure and whole-body CS exposure caused a significant increase in RVSP (*P* < 0.01) and right ventricular hypertrophy index (RVHI, *P* < 0.01) (Fig. 4). Furthermore, RVSP in the nose-only CS exposure group was higher (*P* < 0.05) than that in the whole-body CS exposure group, while RVHI was not (Fig. 4).

### Histological staining and morphological analysis

**Figure 5:**
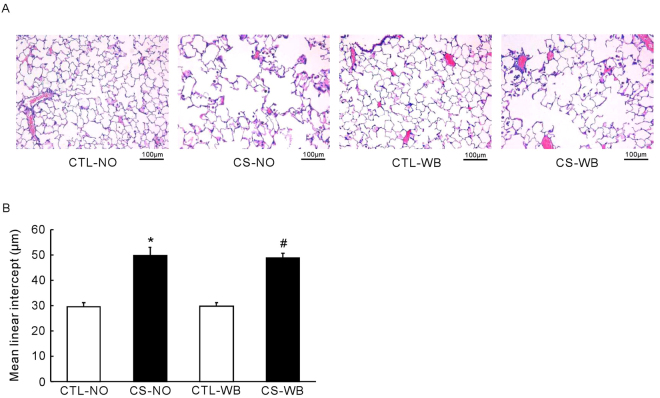
Representative light micrographs of lung tissues of mice (**A**) and the mean linear intercept (**B**), following the group of nose-only CS exposure (CS-NO) or air control (CTL-NO) and the group of whole-body CS exposure (CS-WB) or air control (CTL-WB). Scale bar = 100 μm. Data are presented as mean ± SD (n = 5), *P < 0.01 for CS-NO group vs. CTL-NO group, ^#^ *P* < 0.01 for CS-WB group vs. CTL-WB group.

**Figure 6:**
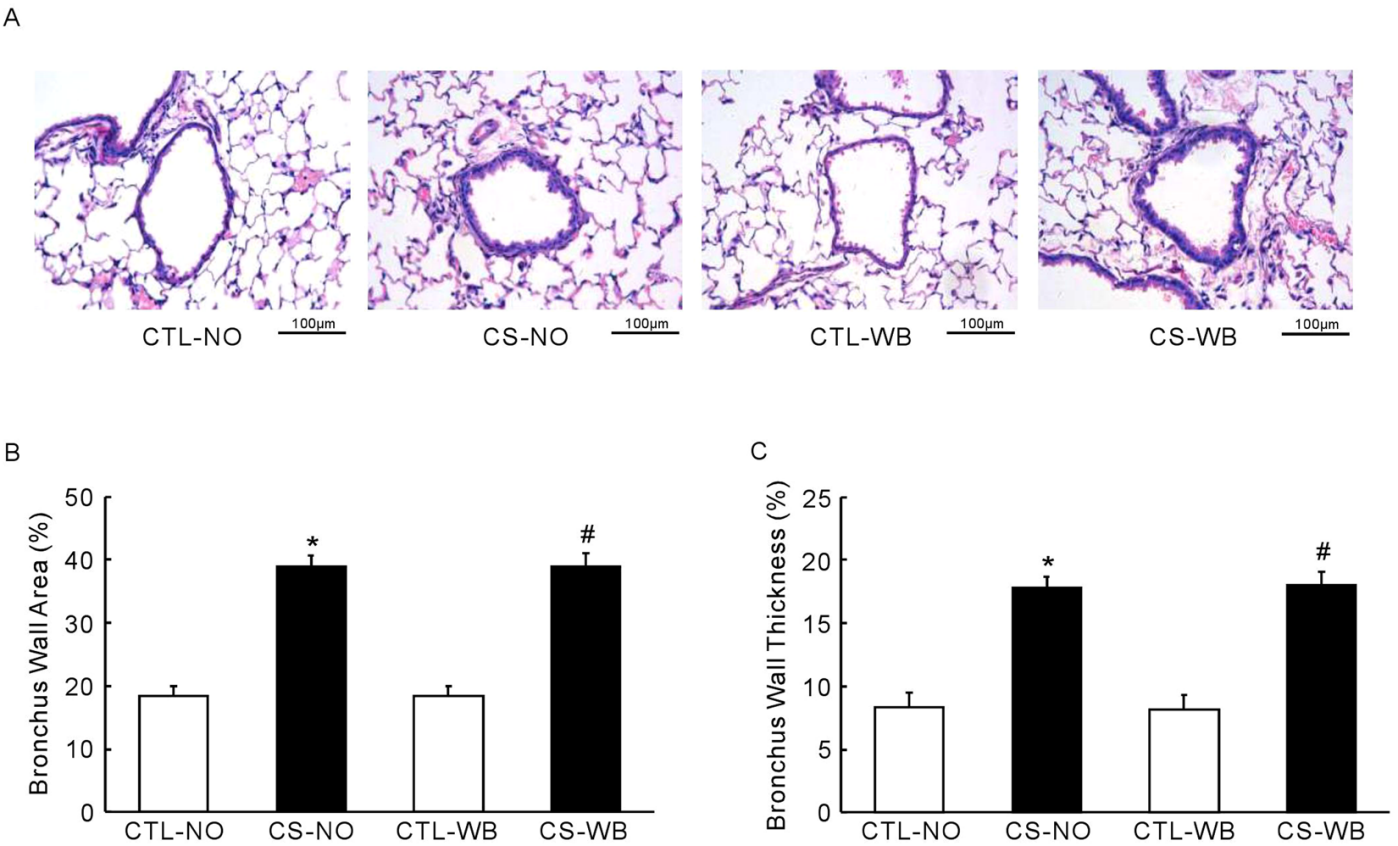
Representative light micrographs of bronchiole of mice (**A**) and the bronchial wall area (**B**) and the bronchial wall thickness (**C**), following the group of nose-only CS exposure (CS-NO) or air control (CTL-NO) and the group of whole-body CS exposure (CS-WB) or air control (CTL-WB). Scale bar = 100 μm. Data are presented as mean ± SD (n = 5), *P < 0.01 for CS-NO group vs. CTL-NO group, ^#^ *P* < 0.01 for CS-WB group vs. CTL-WB group.

**Figure 7:**
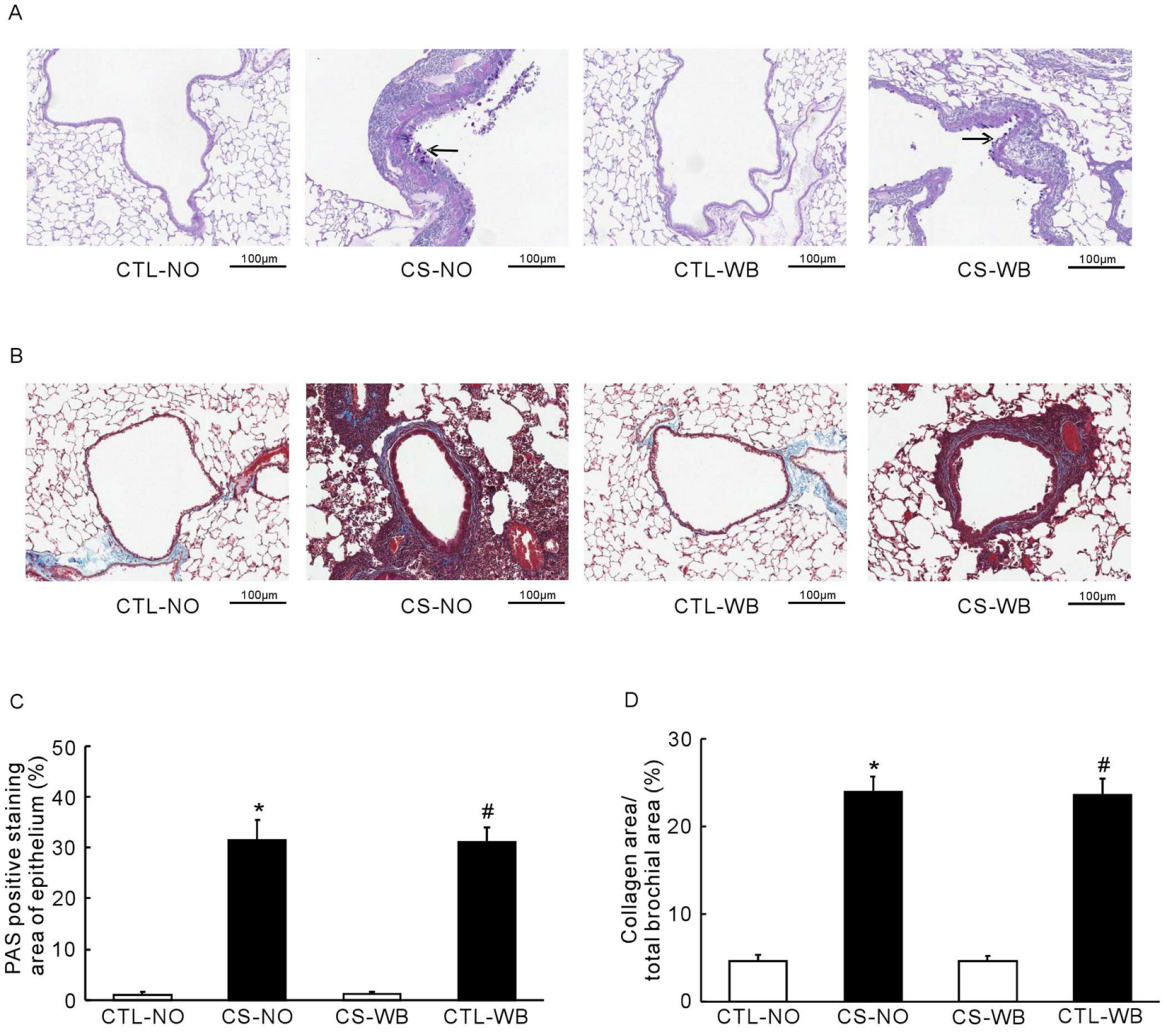
The PAS staining (**A**) and the Masson’s trichrome staining (**B**), the PAS positive staining area of epithelium (**C**) and the ratio of collagen area/total brochial area (**D**), following the group of nose-only CS exposure (CS-NO) or air control (CTL-NO) and the group of whole-body CS exposure (CS-WB) or air control (CTL-WB). Scale bar = 100 μm. Data are presented as mean ± SD (n = 5), *P < 0.01 for CS-NO group vs. CTL-NO group, ^#^ *P* < 0.01 for CS-WB group vs. CTL-WB group.

As is shown in the Figs. 5, 6 and 7, sections of lung from control mice showed a normal appearance under light microscopy. Lung sections from CS-exposed (nose-only and whole-body) mice showed the bronchial walls thickening, the alveolar walls thinning and fracturing, a large number of macrophages and neutrophils interstitial infiltration, and even more pulmonary bullae formation. The PAS staining showed that bronchial walls thickening, goblet cells hyperplasia and inflammatory cells interstitial infiltration in both the nose-only CS exposure group and the whole-body CS exposure group. As shown by the arrow in Fig. 7A, the purple point is the PAS positive staining, showed the goblet cells hyperplasia. At the same time, Masson’s trichrome staining showed that bronchial walls thickening, the collagen fibers were deposited around the airway and a large number of inflammatory cells interstitial infiltration in both the nose-only CS exposure group and the whole-body CS exposure group while compared with their respective control groups. According to the data, the mean linear intercept, the bronchial wall area, the bronchial wall thickness, the PAS positive staining area of epithelium and the ratio of collagen area/total brochial area were significantly increased (*P* < 0.01) in both the nose-only CS exposure group and the whole-body CS exposure group while compared with their respective control groups. However, there was still no significant difference between the nose-only CS exposure group and the whole-body CS exposure group in these indexes above.

**Figure 8:**
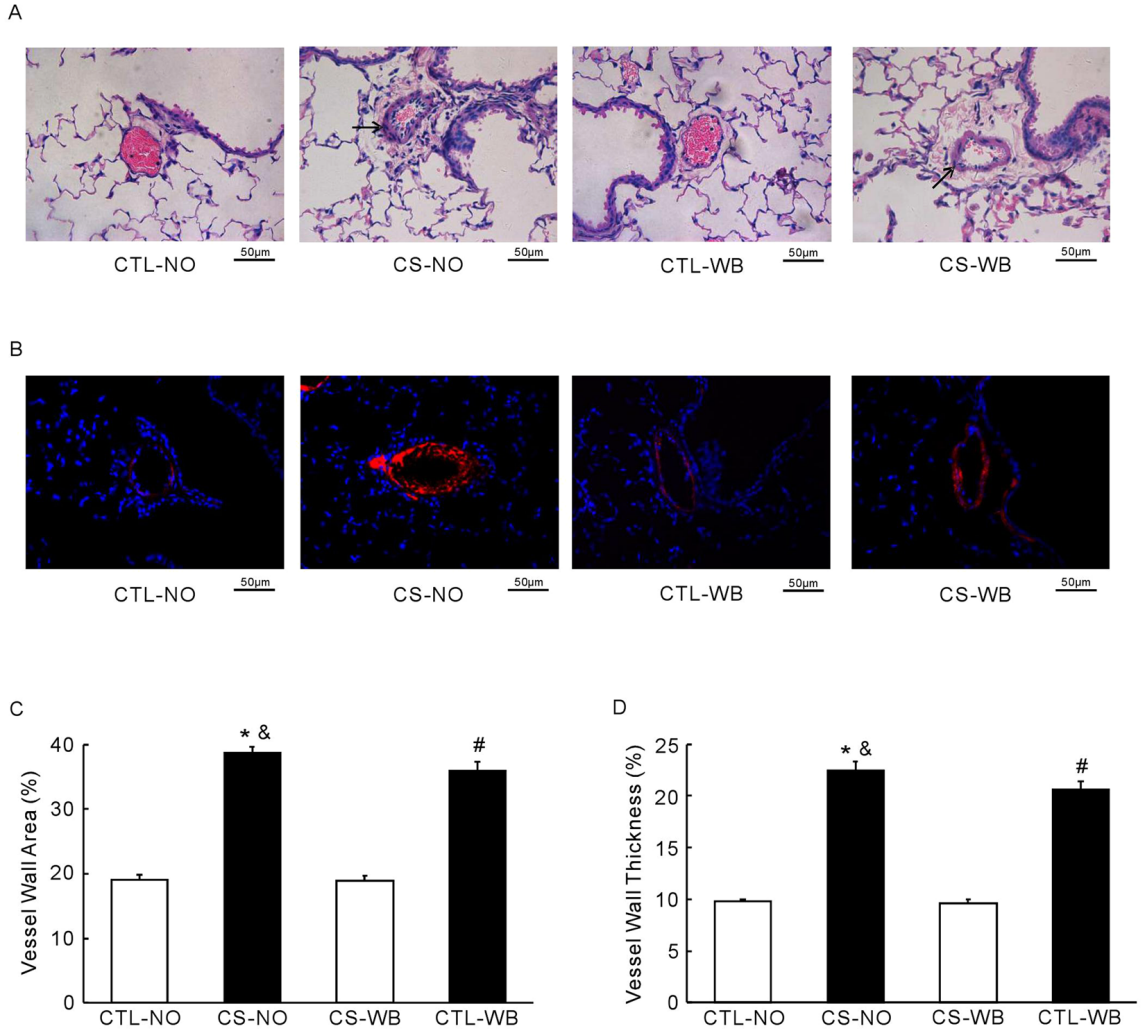
Representative light micrographs of distal small vessels of mouse’s lung (**A**) and the α-SMA staining (**B**), the vessel wall area (**C**), and the vessel wall thickness (D), following the group of nose-only CS exposure (CS-NO) or air control (CTL-NO) and the group of whole-body CS exposure (CS-WB) or air control (CTL-WB). Scale bar = 50 μm. Data are presented as mean ± SD (n = 5), *P < 0.01 for CS-NO group vs. CTL-NO group, ^#^ *P* < 0.01 for CS-WB group vs. CTL-WB group, ^&^ *P* < 0.05 for CS-NO group vs. CS-WB group.

Muscularization of pulmonary vessel was identified by H&E and α-SMA staining. Shown in the Fig. 8, the distal small vessels of lung from air-exposure mice showed a normal appearance under light microscopy. Distal pulmonary small vessels from CS-exposed (nose-only and whole-body) mice showed lymphocytes and neutrophils interstitial infiltration, and the whole layer thicken. According to the data, the vessel wall area and the vessel wall thickness were significantly increased (*P* < 0.01) in both the nose-only CS exposure group and the whole-body CS exposure group, compared with their respective air-exposure groups. In both of these two indexes, the nose-only CS exposure group tested higher (*P* < 0.05) than those in the whole-body CS exposure group. In addition, as shown by the black arrows in Fig. 8A, there are a large number of inflammatory cells around the vasculature, which suggests perivascular inflammation in the distal small vessels of lung, in both the nose-only CS exposure group and whole-body CS exposure group.

### Determination of hematocrit index (HCT) and D-Dimer

**Figure 9:**
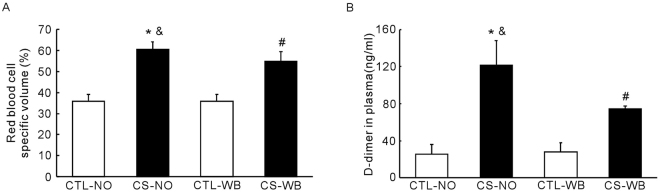
The level of determination of hematocrit index (**A**) and the level of D-dimer in plasma (**B**), following the group of nose-only CS exposure (CS-NO) or air control (CTL-NO) and the group of whole-body CS exposure (CS-WB) or air control (CTL-WB). Data are presented as mean ± SD (n = 6–9), *P < 0.01 for CS-NO group vs. CTL-NO group, ^#^ *P* < 0.01 for CS-WB group vs. CTL-WB group, ^&^ *P* < 0.05 for CS-NO group vs. CS-WB group.

The level of HCT was significantly increased (*P* < 0.01) in the nose-only CS exposure group and the whole-body CS exposure group, compared with their respective air-exposure groups. And the level of HCT was higher (*P* < 0.05) in the nose-only CS exposure group than in the whole-body CS exposure group (Fig. 9A).

The level of D-dimer was significantly increased (*P* < 0.01) in the nose-only CS exposure group and the whole-body CS exposure group, compared with their respective air-exposure groups. And the level of D-dimer was higher (*P* < 0.01) in the nose-only CS exposure group than that in the whole-body CS exposure group (Fig. 9B).

## Discussion

CS exposure is the most appropriate model to study COPD in mice, and there are several exposure systems available^8–10^. The data from our study provides evidence that both the nose-only CS exposure, and the whole-body CS exposure, adding airway LPS inhalation to each, can induce chronic airway inflammation, lung function impairment, emphysema, and right ventricular dysfunction, which are all important disease processes to have present for study, in order to create a complete mouse model. Although the two methods can both be successfully used to build a COPD mouse model, for purposes of experimental testing, accurate measurement of CS absorption through the skin in the whole-body CS exposure system is not possible, while the amount of smoke the mouse inhaled in the nose-only CS exposure model, can be accurately be measured. And so, the nose-only CS exposure system is more controllable and reproducible. Also, in our study, we observed that the smoke residue on the skin of the mice in the whole-body CS exposure system required a longer than expected smoke exposure time, and caused pruritus, malaise, irritability, and fighting in the mice. These problems can be avoided by employing the nose-only exposure system. In addition, there remain a few differences in the right ventricular dysfunction indexes, and the intimal thickening of pulmonary small artery, as well as HCT levels, between these two smoke-intake mice models.

LPS is a major proinflammatory glycolipid component of the gram-negative bacteria cell wall. In our earlier study, we found that the combination of LPS and CS can cause significant changes in airway inflammation and lung function^14^. This is consistent with the causes of COPD, which are mainly cigarette smoke and airway infections. Therefore, the method of combining LPS and CS aimed to simulate the infection-induced acute exacerbation of COPD, which leads to a patient’s sharp decline in pulmonary function. In this study, the mouse model was established by combining cigarette smoke exposure with LPS intranasal inhalation, a method which mimics the development of human COPD, which exhibits bacterial colonization and acute exacerbations^15^. The use of LPS helps to establish a mouse model with emphysema in a shorter time than those relying on only cigarette smoke exposure^16^.

In this study, our data shows that given equal applications of particulate matter, the pulmonary pathological changes associated with COPD in the nose-only exposure mouse models, are relatively similar to the whole-body CS exposure mouse models. The number of total cells in BALF in both the nose-only CS exposure group and the whole-body CS exposure was significantly increased. compared with their respective air-exposure groups. And the proportion of neutrophils and lymphocytes in BALF increased, while the proportion of macrophages decreased, in the two CS exposure groups, compared with the control groups. However, no significant difference was found between the two CS exposure groups in cell composition and number in BALF. This suggests that the mechanism of CS-caused lung damage from nose-only exposure and whole-body CS exposure, may be the same. Generally speaking, cigarette smoke could induce airway constriction and mucus secretion. The particulate matter would deposit in the small airway, which could cause a chronic inflammatory response. And this chronic inflammatory response would destroy the lung parenchyma, take away the alveolar walls, and permanently expand the small airway, which could also induce emphysema and airway remodeling^6,17^. In 2015, the Global Initiative for Chronic Obstructive Lung Disease (GOLD), articulated a new opinion that the more particulate matter a person inhales in life, the more risk of COPD he would have^1^. Our data appears to confirm this new point of view, and suggests that the particulate matter in cigarette smoke may well play an important role in the occurrence and development of COPD.

Another part of our results show that given the same level of particulate matter exposure, the change of right ventricular pressure and the intimal thickening of pulmonary small artery in the nose-only CS exposure mouse model are a little more serious than those in the whole-body CS exposure mouse model. Originally, it was believed that hypoxia, emphysema, and the loss of vascular bed were the primary cause of pathological lung changes associated with COPD, and that increased pulmonary artery pressure was a secondary cause^6^. However, recently it has been shown that inducible nitric oxide synthase (iNOS) is important in the development of pulmonary hypertension following CS exposure^18,19^, while both prostacyclin and endothelial NOS are protective against pulmonary hypertension induced by hypoxia^6,20^. Our team’s previous studies demonstrated that the nicotine in cigarette smoke may induce contraction and proliferation of smooth muscle cells, by directly stimulating pulmonary artery smooth muscle cells (PASMCs), and increasing the levels of intracellular calcium and store-operated calcium entry channels (SOCE) in PASMCs^7,21^. This suggests that chronic CS exposure may lead to pulmonary hypertension, given the presence of pathophysiological mechanisms associated with COPD, and that certain substances in the smoke may have a direct effect on the pulmonary vasculature. At the same time, given the same level of particulate matter application, the measured smoke concentration from nose-only CS exposure was different from that in the whole-body CS exposure system, and the fluctuations in smoke concentration in the nose-only CS exposure system, is similar to the sine wave, successfully mimicing the human smoking situation, while concentrations in the whole-body CS exposure system remained at a stable level. Moreover, there are obvious differences in the concentration of various kinds of substances between direct cigarette smoke and second hand smoke^22^. Therefore, it is possible that the differences in smoke concentration fluctuations and the concentration of substances between the nose-only CS exposure system and the whole-body CS exposure system, are responsible for the derivations observed in right ventricular pressure and the intimal thickening of pulmonary small artery.

And, since there is only a slight increase in right ventricular pressure, and a little more serious in pulmonary vascular damage in the nose-only CS exposure mouse model, compared with the whole-body CS exposure mouse model, then the reason for these slight derivations could be that the amount of time of CS exposure in the current study was not as long as in our previous studies^7,23^.

In addition, we also found that the level of HCT was significantly increased in two model groups, compared with their respective control groups, and that the level of HCT was significantly higher in the nose-only CS exposure group than that of the whole-body CS exposure group. Therefore, we hypothesize that the difference in the HCT levels is related to the difference in right ventricular pressure between the two groups. Firstly, the increase of HCT indicates chronic hypoxia, damaging the lung structure in model animals exposed to long-term CS, and leading to reduced lung ventilation and diffuse function. In order to adapt to chronic hypoxia, the increase of HCT helps enhance the body’s ability to carry oxygen, and chronic hypoxia is also an important factor leading to pulmonary hypertension; Secondly, the increase of HCT will increase blood viscosity resulting in slow blood flow and increased intravascular pressure. In clinical trials, some of the COPD patients got a secondary erythrocytosis. The higher the level of HCT, by increasing blood viscosity, can slow the velocity of blood flow, increase the pulmonary circulation resistance, and format the pulmonary vascular micro thrombus^24,25^. At the same time, some studies suggest that the risk of the pulmonary embolism and other venous thrombosis in COPD patients is double while compared with the non-COPD patients^26^. Thirdly, the increased HCT will increase the risk of intravascular thrombosis formation, and the formation of small pulmonary thrombosis will affect the pulmonary artery pressure. In order to find evidence of thrombosis, we tested the D-dimer in the plasma of the model mice. Since D-dimer reflects the fibrinolytic function, then D-dimer will rise as long as there is thrombosis formation and fibrous dissolution. And our data show that D-dimer was significantly higher in the nose-only CS exposure group than that of the whole-body CS exposure group, suggesting that nose-only CS exposure group had more micro thrombosis, which may lead to more severe pulmonary hypertension.Therefore, we hypothesize that in the COPD mouse model, the secondary erythrocytosis and the formation of pulmonary vascular micro thrombus may be another cause of the rise in pulmonary artery pressure.

In conclusion, we ran parallel whole-body and the nose-only experiments to establish our CS-exposed mouse model. And the results demonstrate that a COPD mouse model can be established by whole-body CS exposure and nose-only CS exposure plus airway LPS inhalation. As mentioned above, the nose-only CS exposure system is more controllable and reproducible. In addition, the model established by nose-only CS exposure has a greater impact on the change of right ventricular pressure and the intimal thickening of pulmonary small artery. Given all that, the nose-only exposure method may be a more suitable way to build the model.

## Materials and Methods

### Animals and CS exposure

Wild-type C57BL/6J mice (6–8 weeks old) were purchased from Nanjing Biomedical Research Institute of Nanjing University (Nanjing, China). Animals were housed in the specific pathogen-free facilities and the Animal Care and Use Committee of Guangzhou Medical University, following all of the approved experimental protocols. In addition, all testing methods were performed in accordance with the guidelines and regulations approved by the Animal Care and Use Committee of Guangzhou Medical University. To establish the COPD mouse model, mice inhaled LPS (7.5 μg in 50 μl saline) at day 0 and day 14. Then the animals were divided into four groups, the nose-only CS exposure (CS-NO) group, the whole-body CS exposure (CS-WB) group, the nose-only control (CTL-NO) group, and the whole-body control (CTL-WB) group. The mice were exposed to cigarette smoke (9 cigarettes each hour, 2 hours each time, twice a day and 6 days per week) in either a whole-body exposure system or in a nose-only exposure system for 10 weeks, excluding the days of LPS injection, and the control mice were exposed to normal air in a respective exposure system. Cigarette used in this study is Plum brand, produced by Guangdong Tobacco Industry CO., Ltd. (Guangzhou, China). Each cigarette yields 11 mg tar, 1.0 mg nicotine and 13 mg CO. In both of these exposure systems, the total particulate density concentration of CS was measured daily and indicated an average of 700 mg total particulate matter per m^3^ (TPM/m^3^), while the concentration of oxygen was (20.5 ± 0.5)%, carbon dioxide was between 4000 to 5000 ppm, and carbon monoxide was between 500 to 800 ppm.

### Lung function measurement

Lung function was measured by using Forced Pulmonary Maneuver System (Buxco Research Systems, Wilmington, North Carolina USA) following the manufacturer’s protocol. Briefly, mice anesthetized with pentobarbital (50 mg/kg body weight in saline) were tracheostomized, intubated and placed in the body chamber of the system. The average breathing frequency was set to 150 breathes/min. Three semiautomatic maneuvers, Boyle’s law, quasi-static pressure volume (PV) and fast flow volume maneuver were performed with the Buxco Systems. Resistance and FRC was determined with Boyle’s law. Chord compliance (C-chord) was measured with quasi-static PV maneuver. Forced expiration volume in 100 milliseconds (FEV_100_) and forced vital capacity (FVC) were recorded by the fast flow volume maneuver.

### Bronchoalveolar lavage fluid collection, cell counting and alveoli intercept measurement

Mouse lung was lavage with 0.6 ml saline/lung for three times. Total cells in bronchoalveolar lavage fluid (BALF) were collected and counted by using a hemocytometer. The cells were then subjected to hematoxylin and eosin (H&E) staining for differential counting of neutrophils, macrophages, and lymphocytes under microscopy. The lung tissues were fixed with 4% paraformaldehyde, embedded with paraffin, sectioned and stained with H&E. Image Analysis Software IPP6.0 was used to assess the mean intercept (Lm) of alveoli, which means the ratio of total length of alveolar to the number of alveoli per field under microscopy. At least three fields per mouse were taken.

### ELISA

The protein levels of Interleukin 6 (IL6) Keratinocyte (KC) in BALF were measured by ELISA. The measurement of IL6 in BALF was performed with IL6 ELISA kits (San Diego, CA). The capture and detection antibodies for KC measurement were obtained from R&D Systems Inc (Minneapolis, MN). Signal was developed by using TMB Substrate Reagent Set (BD Biosciences Pharmingen).

### Hemodynamic measurements

Measurements, including right ventricular pressure and right ventricular hypertrophy, were performed as previously described. Briefly, at the end of chronic cigarette smoke exposure, mice were anesthetized with 1% pentobarbital sodium (70 mg/kg ip). An incision was made in the abdomen, resulting in the visualization of the diaphragm. A heparinized saline-filled 23-gauge needle connected to a pressure transducer was inserted through the diaphragm into the right ventricle (RV), the right ventricular systolic pressure (RVSP) was recorded and measured. The right ventricle was dissected from the left ventricle (LV) and septum (S) after removal of the atria. The ventricles were blotted dry and weighed. Total ventricular weight (g), left ventricular weight (g), and ratio of RV to LV plus S [RV/(LV + S)] were assessed. After pressure recording, whole blood was collected via right ventricle puncture with K2EDTA as an anticoagulant, filled in capillary tubes (0.5 mm outside diameter, VWR Scientific), centrifuged for 5 min at 7,000 rpm, and read on a hematocrit chart (VWR Scientific)

### Histological staining & morphological analysis

After the animals were sacrificed, the left lungs were fixed in 4% paraformaldehyde for 24 hours and then dehydrated and embedded in paraffin, the samples were cut into sections of 4 µm and stained with H&E for general histological examination or stained with periodic acid-Schiff (PAS) for goblet productions determination or Masson’s trichrome to detect fibrosis and collagen deposition according to conventional methods. As previously described, alveolar enlargement and destruction were determined by the mean linear intercept. Briefly, the lines were randomly placed on each of the 10 lung sections, the number of intercepts crossing the lines were counted, and the mean linear intercept was calculated from the length of the lines, multiplied by the number of the lines, divided by the sum of all counted intercepts. To calculate pulmonary arterial wall thickness, the slides were observed and photographed by using a Leica DM4000 B microscope with 20× and 40× objectives. The lumen and total area, as well as the wall thickness and arterial diameter of the 50 to 100 μm (outer diameter) pulmonary arteries were measured using Image-Pro Plus 6.0 software. Similarly, to calculate the thickness of collagen and the bronchial wall, the lumen and total area, as well as the wall thickness, were measured by using Image Pro Plus 6.0 software. The PAS-positive area and total area of corresponding bronchial epithelium were measured. Data was presented as the ratio of PAS-positive area to the total area. Muscularization of pulmonary vessels was identified by H&E and α-SMA staining.

### Determination of hematocrit index and D-dimer

200 μl of blood was extracted from the heart of the mouse by a syringe with heparin sodium and mixed in an Eppendorf tube (EP tube) 100 μl with heparin sodium (125 IU/ml concentration). Then, blood was drawn into a capillary tube and then put onto a capillary high-speed centrifuge (Parameter setting: room temperature, 5000 rpm, and 10 min). The hematocrit index can be read after centrifugation.

600 μl of blood was extracted from the heart of the mouse by a syringe and mixed in an EP tube 100 μl with ethylenediaminetetraacetic acid (EDTA). Then the EP tubes were put into a high-speed centrifuge (Parameter setting: 4 °C, 800 rcf, and 10 min). The plasma was extracted and measured the level of D-dimer, with the mouse D-dimer Elisa kit (CUSABIO).

### Statistical analysis

All experiments were conducted in triplicate and repeated three times. Statistics were analyzed with ANOVA. If significant *F*-ratios were obtained with ANOVA, pairwise comparison of means was conducted with Bonferroni analysis. Data are presented as mean ± SEM. N means the number of experiments repeats in the animals. *P* < 0.05 and *P* < 0.01 were considered as significant and extremely significant, respectively.
